# Induction of plasminogen activator inhibitor type-1 (PAI-1) by hypoxia and irradiation in human head and neck carcinoma cell lines

**DOI:** 10.1186/1471-2407-7-143

**Published:** 2007-07-30

**Authors:** Daniela Schilling, Christine Bayer, Anneke Geurts-Moespot, Fred CGJ Sweep, Martin Pruschy, Karin Mengele, Lisa D Sprague, Michael Molls

**Affiliations:** 1 Department of Radiation Oncology, Klinikum rechts der Isar, Technical University of Munich, Ismaninger Str. 22, 81675 Munich, Germany; 2 GSF – Institute of Pathology, KKG, Innate Immunity in Tumor Biology, Ingolstädter Landstraße 1, 85764 Neuherberg, Germany; 3 Department of Chemical Endocrinology, Radboud University Nijmegen Medical Centre, Geert Grooteplein 8, 6500 HB Nijmegen, The Netherlands; 4 Department of Radiation Oncology, University Hospital Zürich, Ramistr. 100, 8091 Zürich, Switzerland; 5 Clinical Research Unit of the Department of Obstetrics and Gynaecology, Klinikum rechts der Isar, Technical University of Munich, Ismaninger Str. 22, 81675 München, Germany; 6 Institute of Molecular Pathogenesis, Friedrich-Loeffler-Institut, Naumburgerstr. 96a, 07743 Jena, Germany

## Abstract

**Background:**

Squamous cell carcinoma of the head and neck (SCCHN) often contain highly radioresistant hypoxic regions, nonetheless, radiotherapy is a common treatment modality for these tumours. Reoxygenation during fractionated radiotherapy is desired to render these hypoxic tumour regions more radiosensitive. Hypoxia additionally leads to up-regulation of PAI-1, a protein involved in tumour progression and an established prognostic marker for poor outcome. However, the impact of reoxygenation and radiation on PAI-1 levels is not yet clear. Therefore, we investigated the kinetics of PAI-1 expression and secretion after hypoxia and reoxygenation, and determined the influence of ionizing radiation on PAI-1 levels in the two human SCCHN cell lines, BHY and FaDu.

**Methods:**

HIF-1α immunoblot was used to visualize the degree of hypoxia in the two cell lines. Cellular PAI-1 expression was investigated by immunofluorescence microscopy. ELISA was used to quantify relative changes in PAI-1 expression (cell lysates) and secretion (cell culture supernatants) in response to various lengths (2 – 4 h) of hypoxic exposure (< 0.66 % O_2_), reoxygenation (24 h, 20 % O_2_), and radiation (0, 2, 5 and 10 Gy).

**Results:**

HIF-1α expression was induced between 2 and 24 h of hypoxic exposure. Intracellular PAI-1 expression was significantly increased in BHY and FaDu cells as early as 4 h after hypoxic exposure. A significant induction in secreted PAI-1 was seen after 12 to 24 h (BHY) and 8 to 24 h (FaDu) hypoxia, as compared to the normoxic control. A 24 h reoxygenation period caused significantly less PAI-1 secretion than a 24 h hypoxia period in FaDu cells. Irradiation led to an up-regulation of PAI-1 expression and secretion in both, BHY and FaDu cells.

**Conclusion:**

Our data suggest that both, short-term (~4 – 8 h) and long-term (~20 – 24 h) hypoxic exposure could increase PAI-1 levels in SCCHN *in vivo*. Importantly, radiation itself could lead to PAI-1 up-regulation in head and neck tumours, whereas reoxygenation of hypoxic tumour cells during fractionated radiotherapy could counteract the increased PAI-1 levels.

## Background

The adverse effects of tumour hypoxia on the outcome after radiotherapy are well established for many different tumour types and in particular for SCCHN [1-4]. This is in part due to the oxygen effect; hypoxic cells require a 2.5 to 3-fold higher radiation dose to achieve the same cell kill seen in well-oxygenated cells. Therefore, reoxygenation of hypoxic tumours during fractionated radiotherapy is intended to render tumour cells more radiosensitive. In addition, hypoxia causes a differential expression profile which can lead to a biologically more aggressive and radioresistant phenotype.

One important protein that is up-regulated under hypoxic conditions is the plasminogen activator inhibitor type-1 (PAI-1), a multifunctional protein, best known for its role as inhibitor of urokinase-type plasminogen activator (uPA) [5,6]. The activated serine protease uPA is able to convert plasminogen to plasmin, which in turn degrades the extracellular matrix (ECM) and leads to invasion and metastasis of tumour cells. Despite its inhibiting function of uPA, numerous clinical studies demonstrate a strong correlation between high tissue levels of PAI-1 and poor prognosis for several tumour entities [7], including squamous cell carcinomas of the head and neck [8,9]. This paradoxical finding might be explained by further biological functions of PAI-1 in promoting migration and angiogenesis and in inhibiting apoptosis of tumour cells [10-13].

Previous studies have shown that both hypoxia inducible factors, HIF-1 and HIF-2, are able to transcriptionally up-regulate human PAI-1 via binding to the hypoxia responsive element (HRE) within the PAI-1 promoter region [14,15]. An increase in PAI-1 mRNA has been observed *in vitro *as early as 2 h after hypoxic exposure in SCCHN, human hepatoma and lung cancer cell lines [14,16,17]. Only one detailed time-course analysis of cellular PAI-1 expression levels after hypoxic exposure exists and was performed on a single human hepatoma cell line [14]. Up-regulation of PAI-1 protein secretion by low levels of oxygen has also been demonstrated in various human cell lines, but so far not in SCCHN [14,16,18,19]. Furthermore, very few studies – and none on SCCHN – exist investigating whether radiation can lead to enhanced PAI-1 levels [20-22].

As PAI-1 is a prognostic marker for poor outcome and is involved in tumour progression, a better understanding of the factors (e.g., hypoxia and irradiation) responsible for PAI-1 up-regulation in SCCHN is vital for treatment planning of patients with these especially hypoxic and radioresistant tumours. Therefore, the aim of our study was first to investigate the impact of the length of hypoxic exposure and reoxygenation on PAI-1 expression and secretion in the two human SCCHN cell lines, BHY and FaDu. Secondly, we examined the influence of ionizing radiation on PAI-1 expression and secretion in these same two cell lines.

## Methods

### Cell lines

The two human cell lines, BHY and FaDu, were used in this study. BHY derives from a highly differentiated and invasive SCC from the lower alveolus (DSMZ ACC 404) [23] and FaDu originates from an undifferentiated hypopharyngeal SCC (ATCC HTB-43) [24]. Cells were cultured in Dulbecco's Modified Eagle's Medium (D-MEM) (Invitrogen) containing 10 % foetal calf serum (FCS), 0.05 mg/ml penicillin, 0.05 mg/ml streptomycin and 0.1 mg/ml neomycin and maintained in a humidified atmosphere of 95 % air and 5 % CO_2 _at 37°C (standard conditions).

### Hypoxic cultivation of cells

For hypoxia experiments, 5 × 10^5 ^cells were seeded into 10 cm cell culture dishes. After 72 h pre-incubation under standard conditions, the medium was changed and the dishes were incubated for various lengths of time (2, 4, 8, 12, 16, 20, 24 h) under normoxic (= standard conditions) or hypoxic conditions at 37°C. To achieve hypoxic conditions, dishes were placed into airtight aluminium chambers connected to a vacuum pump and a N_2 _gas cylinder. By alternating eleven times between oxygen evacuation and N_2 _inflow, an oxygen concentration below 0.66 % (5 mm Hg) was reached after 22 min.

For reoxygenation studies, cells were removed from the aluminium chambers after 24 h of hypoxia, then the medium was changed and cells were cultivated for an additional 24 h under normoxic conditions (24hH/24hN). As a control, parallel samples were incubated for 24 h under normoxia, then the medium was changed and cells were incubated further for 24 h under normoxic conditions (24hN/24hN).

In addition, after 24 h of hypoxia, cell-free supernatants were transferred to fresh 10 cm cell culture dishes and incubated for 1, 2, 3 and 7 days under normoxic conditions.

### Trypan blue exclusion

This assay was used to determine the ratio of viable cells. Cells cultivated 24 h under normoxia or hypoxia were trypsinized, centrifuged, resuspended in medium, and mixed with an equal volume of 0.4 % trypan blue staining solution. Viable and dead cells were counted using a haemocytometer.

### Irradiation

For irradiation experiments, 5 × 10^5 ^cells were seeded into 10 cm cell culture dishes. After 72 h pre-incubation under standard conditions, the medium was changed and cells were irradiated with 2, 5 and 10 Gy using the RT100 irradiation device (Philips) at a dose rate of ~1 Gy/min. Irradiations were performed at room temperature under atmospheric conditions. Control cells received sham irradiation (= 0 Gy). After irradiation, cells were cultivated for 45 h under standard conditions.

### ELISA

Supernatants of cultured cells were centrifuged to remove debris and stored at -20°C. Attached cells were washed twice with cold PBS and lysed in 1 × TBST buffer (0.05 M Tris/HCl, pH 8.5, 0.1 M NaCl, 10 mM EDTA, 0.5 % Tween20 and 0.1 % Triton X-100). The cell suspension was rotated for 24 h at 4°C, centrifuged and the supernatant (= cell lysate) was stored at -20°C until further use. Protein content in the cell lysates was determined using the BCA™ Protein Assay Kit (Pierce). PAI-1 concentrations in the cell lysates and supernatants were determined by ELISA as described previously [25]. All measurements were performed in duplicate. The mean values and standard errors of the mean (S.E.M.) were calculated from at least three independent experiments.

For hypoxia and reoxygenation experiments, the PAI-1 antigen content in cell lysates and supernatants was calculated relative to the total protein content in the corresponding cell lysate. For the calculation of relative PAI-1 levels, the PAI-1 concentrations in either cell lysates or supernatants at 24 h normoxia were set to one.

For irradiation experiments, the PAI-1 antigen concentration in cell lysates and supernatants was calculated relative to the viable adherent cells at the time point of sample collection. For the calculation of relative PAI-1 levels, PAI-1 concentrations in the unirradiated samples (0 Gy) were set to one.

### Western Blot

Cell lysates (5 μg) were separated by SDS-polyacrylamide gel electrophoresis (10 %) and blotted onto a PVDF membrane. Membranes were blocked 1 h at RT with 5 % non-fat dry milk and 0.1 % Tween-20 in PBS. Subsequently, membranes were incubated with monoclonal anti- HIF-1α antibody (BD Biosciences Pharmingen, 1:250) or monoclonal anti-β-actin antibody (Sigma, 1:20000) at 4°C overnight. After washing, the blots were incubated with secondary goat anti-mouse IgG horseradish peroxidase conjugate (Promega, 1:2500) for 1 h at RT. Detection was performed with self-made ECL (1.25 mM Luminol, 0.2 mM p-Coumaric acid, 0.09% H_2_O_2_, 100 mM Tris/HCl pH 8.6). Cells treated with 100 μM desferrioxamine (DFO) for 24 h under standard conditions served as positive controls.

### Immunofluorescence

For immunofluorescence studies, 5 × 10^5 ^cells were seeded onto 3-well diagnostic microscope slides (Erie Scientific Company) in 10 cm cell culture dishes. After 72 h, the medium was exchanged and cells were exposed to normoxic or hypoxic conditions. Afterwards, cells were fixed with 4 % paraformaldehyde, permeabilized with saponin (0.025 %; 45 min at RT) and subsequently incubated in 2 % normal goat serum to block unspecific binding. Rabbit anti-PAI-1 antibody (PAb-Rb) [25] was diluted 1:125 (FaDu) or 1:250 (BHY) in 0.1 % BSA-PBS and applied to the slides (4°C, O/N). Cells were stained in the dark for 30 min with Alexa Fluor^® ^488 goat anti-rabbit IgG (Invitrogen) (1:400) to visualize PAI-1 and Alexa Fluor^® ^568 phalloidin (Invitrogen) (1:100) to visualize the cytoskeleton. Slides were then washed with PBS and mounted with VECTASHIELD^® ^Mounting Medium containing DAPI (Vector Laboratories). Fluorescence was observed using an Axioskop 2 plus fluorescence microscope (Zeiss) and the appropriate filters (Chroma Technology, BFIOptilas). Photographic documentation was carried out using an AxioCam MRc5 camera, an Achroplan 100 × oil objective and the AxioVision 4.4 software (Zeiss).

### Statistics

Statistical analysis was performed using SPSS 12.0.1 software. Student's t-test was used to evaluate the differences between normoxic, hypoxic and reoxygenated and between unirradiated and irradiated samples (*p ≤ 0.05, **p ≤ 0.01, ***p ≤ 0.001). Pearson's correlation coefficient r was used to determine linear correlations between intracellular and secreted PAI-1 levels.

## Results

### Hypoxia induces HIF-1α expression

**Figure 1:**
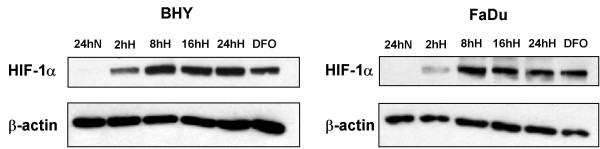
**HIF-1α expression in BHY and FaDu cell lysates**. Cells were maintained under normoxia for 24 h (24hN) or hypoxia for 2 (2hH), 8 (8hH), 16 (16hH) and 24 h (24hH). Cells treated with 100 μM desferroxamine (DFO) for 24 h under normoxic conditions served as positive controls. 5 μg of each cell lysate was loaded per well and visualized on western blots using HIF-1α or β-actin antibodies as indicated.

Since HIF-1α (hypoxia inducible factor-1α) expression is known to increase after hypoxic exposure, HIF-1α was used to validate and visualize the hypoxic induction of cell metabolism in our system. Cells were incubated under normoxic (24 h) or hypoxic (2, 8, 16 and 24 h) conditions and cell lysates prepared. As a positive control, cells were incubated for 24 h with 100 μM desferroxamine (DFO) under aerobic conditions. Detection of HIF-1α by immunoblotting shows HIF-1α expression both under hypoxic conditions (2 – 24 h) and in DFO-treated BHY and FaDu cells, but not under normoxic conditions (Fig. 1). HIF-1α levels increase in a time-dependent manner up to 8 h. From 8 h to 24 h, HIF-1α levels remain nearly constant. This indicates that effective intracellular hypoxia was reached as early as 2 h after hypoxic incubation, increased up to 8 h and was stable for at least 24 h.

### Influence of hypoxia on cell viability

Before examining the effects of hypoxia on PAI-1 expression by BHY and FaDu cells, it was necessary to prove whether they could tolerate prolonged periods (24 h) under hypoxic conditions. The counting of cells in the cell culture supernatants revealed that 24 h hypoxia caused a significant detachment of cells (BHY: 29.0 %; FaDu: 13.9 %) (Table 1). Additionally, since PAI-1 concentrations in later ELISA experiments were measured in the cell lysates of adherent cells, it was essential to prove whether 24 h of hypoxia had an effect on the viability of the adherent cells. The determination of viability of the trypsinized adherent cells by the trypan blue exclusion assay demonstrated only a slight and non-significant decrease in viability after 24 h hypoxic exposure (BHY: 97.1 %; FaDu: 98.7 %) compared to normoxic conditions (BHY: 99.1 %; FaDu: 99.4 %) (Table 1).

**Table 1:** Viability of BHY and FaDu cells.

	**% detached cells**		**% viable adherent cells**	
	**24 h N**	**24 h H**	**24 h N**	**24 h H**
**BHY**	3.32 (± 0.57)	29.0 (± 2.36) ***	99.1 (± 0.35)	97.1 (± 1.24)
**FaDu**	0.73 (± 0.03)	13.9 (± 1.66) **	99.4 (± 0.25)	98.7 (± 0.53)

After 24 h of normoxic or hypoxic incubation, detached cells in the supernatants and trypsinized adherent cells were counted and the percentage of detached cells calculated. The percentage of viable adherent cells was determined by trypan blue exclusion assay. Mean values ± S.E.M. of at least four independent experiments are shown. Associated significance of hypoxic vs. normoxic cells is indicated (*p ≤ 0.05, **p ≤ 0.01, ***p ≤ 0.001).

Therefore, hypoxic exposure (24 h) causes increased detachment of cells, but does not lead to significant loss in cell viability of the remaining adherent cells.

### Influence of hypoxia on cell morphology and PAI-1 expression

**Figure 2:**
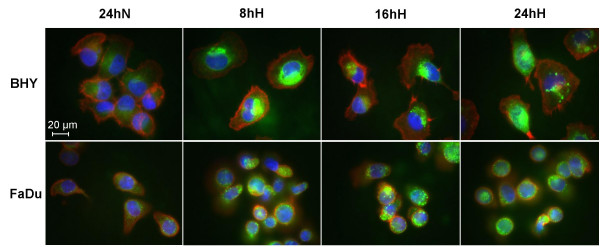
**Influence of hypoxia on cell morphology and PAI-1 expression in BHY and FaDu cells**. Immunofluorescent staining with the PAI-1 antibody, PAb-Rb (BHY 1:250, FaDu 1:125) (green), Phalloidin (red) and DAPI (blue) of BHY and FaDu cells grown for 24 h under normoxic (24hN) and for 8 (8hH), 16 (16hH) and 24 h (24hH) under hypoxic conditions. Images of green fluorescence were consistently exposed for 600 ms for BHY and 900 ms for FaDu.

In order to observe cell morphology and PAI-1 expression under hypoxia, immunofluorescence studies were performed. Cells were grown on slides, exposed to normoxic or hypoxic conditions and stained with an anti-PAI-1 antibody (PAb-Rb) and phalloidin. Staining with phalloidin (red) revealed no significant changes in the cytoskeleton during hypoxic incubation (Fig. 2). PAI-1 detection by PAb-Rb showed a strong green fluorescent signal in the cytoplasm after 8, 16 and 24 h of hypoxia in both cell lines, whereas the fluorescence of the normoxic control remained weak (Fig. 2). This demonstrates that hypoxia induces intracellular up-regulation of PAI-1 in BHY and FaDu cells.

### Kinetics of cellular PAI-1 expression under hypoxia

**Figure 3:**
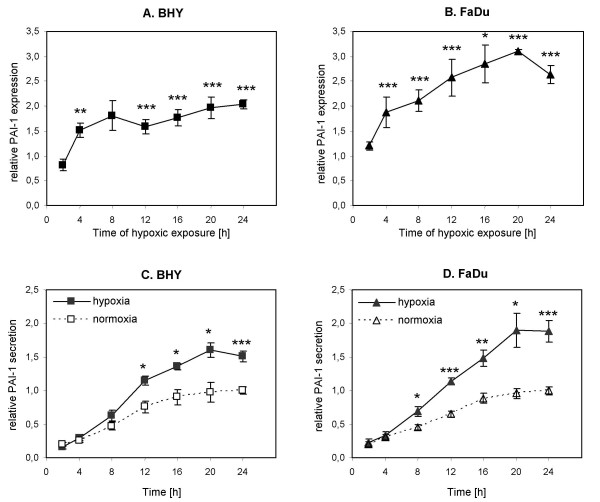
**Kinetics of PAI-1 expression and secretion under hypoxia**. **A, B: **Quantitative determination of PAI-1 levels in BHY (A) and FaDu (B) cell lysates by ELISA. Cells incubated 24 h under normoxia were chosen as the normoxic standards for PAI-1 expression studies and hypoxic values were calculated relative to these values (BHY: 33.5 ng/mg protein; FaDu: 8.6 ng/mg protein). Mean values ± S.E.M. of four independent experiments, each analyzed in duplicate, are shown. Associated significance of the hypoxic samples *vs*. the normoxic standard is indicated (*p ≤ 0.05, **p ≤ 0.01, ***p ≤ 0.001). **C, D: **Quantitative determination of PAI-1 secretion levels in cell culture supernatants from BHY (C) and FaDu (D) cells by ELISA. The PAI-1 secretion level after 24 h normoxia was chosen as the normoxic standard (= 1) and all other values were calculated relative to this value (BHY: 706.5 ng/mg protein; FaDu: 112.8 ng/mg protein). Mean values ± S.E.M. of at least four independent experiments, each analyzed in duplicate, are shown. Associated significance between the hypoxic sample and the corresponding normoxic sample (2 – 24 h) is indicated (*p ≤ 0.05, **p ≤ 0.01, ***p ≤ 0.001).

To investigate the kinetics of PAI-1 expression under hypoxic conditions, BHY and FaDu cells were exposed either to normoxia for 2 and 24 h or to hypoxia for 2, 4, 8, 12, 16, 20 and 24 h. Subsequently, the intracellular PAI-1 levels were determined in cell lysates by ELISA. There was no significant difference in PAI-1 expression between 2 h and 24 h of normoxic incubation in BHY and FaDu cells. Therefore, cells incubated 24 h under normoxia were chosen as the standard for PAI-1 expression studies and hypoxic values were calculated relative to this value. A detailed kinetic analysis of hypoxia revealed a time-dependent increase in PAI-1 expression in BHY cells, starting after 4 h and reaching maximal levels after 24 h of hypoxic exposure (Fig. 3A). FaDu cells showed the same tendency with significantly enhanced PAI-1 expression levels after 4 h, and maximal PAI-1 expression levels after 20 h of hypoxia (Fig. 3B). The maximal relative induction of PAI-1 is 2.0-fold for BHY after 24 h hypoxia and 3.1-fold for FaDu after 20 h hypoxia.

### Kinetics of PAI-1 secretion under hypoxia and normoxia

For the investigation of secreted PAI-1 levels, cells were exposed to the same normoxic and hypoxic conditions as for cellular expression studies. After 2, 4, 8, 12, 16, 20 and 24 h, PAI-1 concentrations in the cell culture supernatants were measured. Importantly, an examination of FCS-containing D-MEM medium (without cells) revealed no detectable PAI-1.

Due to an increase in basal PAI-1 secretion levels from 2 to 24 h normoxic incubation (BHY: 5.0-fold; FaDu: 4.8-fold), PAI-1 kinetics were studied not only under hypoxia but also under normoxia. As shown in figure 3C and 3D, the amount of PAI-1 in the supernatants of both cell lines rises with increasing lengths of both normoxic and hypoxic exposure. A significant difference in PAI-1 secretion between normoxia and hypoxia exists from 12 to 24 h for BHY and from 8 to 24 h for FaDu. The maximal difference between normoxic and hypoxic PAI-1 secretion was seen after 20 h for both cell lines (BHY: 1.7-fold; FaDu: 2.0-fold).

Under hypoxia, a similar time-dependent trend of intracellular (Fig. 3A and 3B) and secreted (Fig. 3C and 3D) PAI-1 levels exists for both cell lines. Accordingly, calculation of the Pearson's correlation coefficient, r, revealed a significant correlation between PAI-1 expression and secretion under hypoxia for BHY (r = 0.80, p ≤ 0.05) as well as FaDu (r = 0.91, p ≤ 0.01) cells.

### Effect of reoxygenation on PAI-1 expression and secretion

First, we investigated whether secreted PAI-1 in cell culture medium is detectable by ELISA after longer incubation periods at 37°C. This was important for reoxygenation studies, since we intended to measure only PAI-1 that was secreted during the 24 h reoxygenation period and not during the preceding hypoxic exposure. For this, BHY and FaDu cells were exposed to hypoxic conditions for 24 h. Subsequently, the supernatants were centrifuged to remove detached cells and the cell-free supernatants were further incubated under standard conditions at 37°C. At different time points (0, 1, 2, 3 and 7 days), samples were drawn and PAI-1 concentrations in the cell-free supernatants were determined by ELISA. The PAI-1 levels in these cell-free supernatants remained constant for up to 7 days, indicating that PAI-1 in the medium is detectable by ELISA after prolonged incubation periods at 37°C (Table 2).

**Table 2:** Detection of PAI-1 in cell-free supernatants.

	**PAI-1 concentration [ng/ml]**	
**Incubation time [d]**	**BHY**	**FaDu**
**0**	66.3 (± 5.9)	9.8 (± 1.9)
**1**	66.8 (± 6.4)	10.5 (± 2.8)
**2**	61.0 (± 5.5)	10.2 (± 1.8)
**3**	66.9 (± 4.6)	9.8 (± 2.0)
**7**	73.3 (± 4.3)	10.4 (± 1.8)

Cells were maintained for 24 h under hypoxic conditions, then the cell-free supernatants were incubated at 37°C under standard conditions for up to 7 days. PAI-1 concentrations [ng/ml] ± S.E.M. of three independent experiments, each analyzed in duplicate by ELISA are shown.

Therefore, we decided to change the medium directly before reoxygenation. This ensured that only newly secreted PAI-1 protein during the 24 h reoxygenation period was measured. Two parallel dishes were exposed to hypoxia and two to normoxia for 24 h. Afterwards, cell lysates and supernatants from one of the hypoxic (= 24hH) and one of the normoxic (= 24hN) dishes were prepared or isolated, respectively. In the remaining two dishes, the medium was changed (/indicates the medium change) and cells were kept for an additional 24 h under normoxic conditions (= 24hH/24hN and 24hN/24hN, respectively). In other words, 24hH/24hN is the reoxygenated sample and 24hN/24hN is the normoxic control for medium changed dishes.

**Figure 4:**
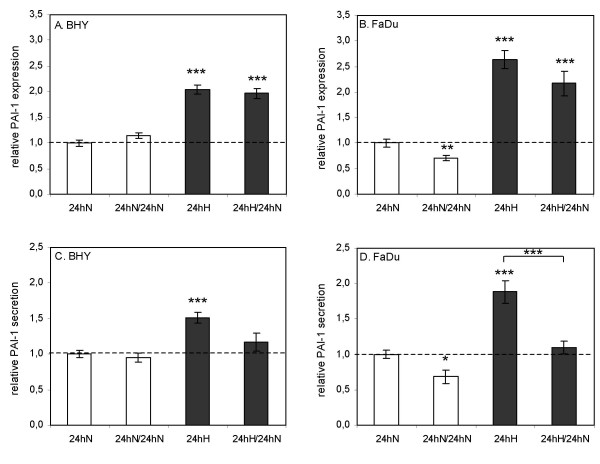
**Effect of reoxygenation on PAI-1 expression and secretion**. PAI-1 concentrations in cell lysates (A and B) and cell culture supernatants (C and D) of BHY and FaDu cells were determined by ELISA. Two parallel dishes were exposed to hypoxic conditions (■) and two to normoxic (□) conditions for 24 h. Subsequently, cell lysates and supernatants from one hypoxic (= 24hH) and one normoxic (= 24hN) dish were prepared or isolated, respectively. In the remaining two dishes, the medium was changed (/indicates medium change) and cells were further kept for 24 h under normoxic conditions (= 24hH/24hN and 24hN/24hN, respectively). Mean values ± S.E.M. of at least four independent experiments, each analyzed in duplicate, are shown relative to the mean normoxic value at the time of medium change (24hN). Associated significance of each sample *vs*. the normoxic standard (24hN) is indicated (*p ≤ 0.05, **p ≤ 0.01, ***p ≤ 0.001). Additionally, the significance of the reoxygenated sample (24hH/24hN) vs. the hypoxic sample (24hH) is indicated in D.

Measurements of PAI-1 concentrations in the cell lysates of BHY and FaDu cells revealed significantly higher PAI-1 expression levels in hypoxic (24hH) and reoxygenated (24hH/24hN) than in normoxic (24hN) cells (Fig. 4A and 4B). The PAI-1 expression in reoxygenated BHY and FaDu cells (24hH/24hN) was slightly less compared to hypoxic cells (24hH).

The supernatants of hypoxic BHY and FaDu cells (24hH) also revealed significantly increased PAI-1 levels compared to normoxic cells (24hN) (Fig. 4C and 4D). During the 24 h reoxygenation period (24hH/24hN), less PAI-1 was secreted than during the 24 h hypoxic incubation period (24hH), which was significant for FaDu cells. The normoxic controls for the medium changed dishes (24hN/24hN) also showed slightly less PAI-1 secretion compared to the 24hN value, however to a lesser extent, indicating a net reduction in PAI-1 release during reoxygenation. These data demonstrate that although marginally more PAI-1 is secreted during reoxygenation than under normoxia, clearly less is secreted than under hypoxia.

### Irradiation induces PAI-1 expression and secretion

**Figure 5:**
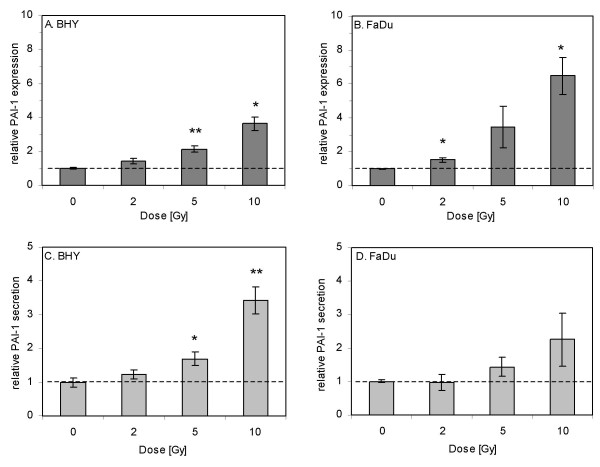
**PAI-1 expression and secretion after irradiation**. 45 h after irradiation with 2, 5 and 10 Gy, PAI-1 concentrations in the cell lysates (A and B) and cell culture supernatants (C and D) of BHY and FaDu cells were determined by ELISA. Unirradiated cells (0 Gy) served as a control. Mean values ± S.E.M. of three independent experiments, each analyzed in duplicate, are shown relative to the 0 Gy mean value. Associated significance of the irradiated samples *vs*. the unirradiated control (0 Gy) is indicated (*p ≤ 0.05, **p ≤ 0.01, ***p ≤ 0.001).

To prove whether irradiation has an effect on PAI-1 expression and secretion, BHY and FaDu cells were irradiated with 0, 2, 5 and 10 Gy under standard conditions. Measurement of PAI-1 concentrations relative to the 0 Gy control in the cell lysates and supernatants 45 h after irradiation showed that both, PAI-1 expression and secretion, rises with increasing dose in both investigated cell lines (Fig. 5). Cellular PAI-1 levels were significantly increased after irradiation with 5 and 10 Gy in BHY cells and with 2 and 10 Gy in FaDu cells (Fig. 5A and 5B). Maximal induction of PAI-1 expression was 3.6-fold for BHY and 6.5-fold for FaDu cells after irradiation with 10 Gy.

PAI-1 concentrations in the cell supernatants were also enhanced 45 h after irradiation (5 and 10 Gy) (Fig. 5C and 5D), but only significantly for BHY cells. Maximal up-regulation of PAI-1 secretion was seen after irradiation with 10 Gy (BHY: 3.4-fold, FaDu: 2.3-fold).

## Discussion

As PAI-1 is involved in the invasion and migration of tumour cells and is an established marker for poor outcome, it is crucial to investigate the exact mechanisms leading to increased PAI-1 levels. Although previous studies have shown that both, hypoxia and irradiation are able to up-regulate PAI-1 in different cell lines, no study has investigated the influence of hypoxia, reoxygenation and irradiation on PAI-1 expression and secretion in the frequently hypoxic tumour entity, SCCHN. Therefore, in the present study, we investigated the influence of these parameters on PAI-1 expression and secretion in the two SCCHN cell lines, BHY and FaDu. Furthermore, we analyzed in detail the kinetics of PAI-1 induction by hypoxia.

The only data available so far on hypoxia kinetics for SCCHN cell lines is by Koong *et al*. [17]. However, they only investigated one cell line (FaDu) on the transcriptional and not on the protein level. They found that PAI-1 mRNA levels were enhanced already 2 h after hypoxic exposure and increased continuously up to 24 h.

Concerning cellular PAI-1 protein levels, we showed significantly increased PAI-1 levels during hypoxic exposure between 4 and 24 h for BHY and FaDu cells (Fig. 3A and 3B). The only study describing detailed kinetics of cell-associated PAI-1 levels under hypoxia demonstrated a similar trend with significantly increased cellular PAI-1 levels between 8 and 48 h in the human hepatoma cell line, HepG2 [14].

Regarding secreted PAI-1 levels, a significant difference in PAI-1 secretion between normoxic and hypoxic cells began after 12 (BHY) or 8 h (FaDu) of incubation and lasted for up to 24 h (Fig. 3C and 3D). In accordance with our results, Kimura *et al*. [16] demonstrated significant up-regulation of PAI-1 secretion after 8 and up to 24 h of hypoxic exposure for a human lung cancer cell line. Similar time-dependent studies on transformed and non-transformed human and mouse cell lines showed significantly elevated PAI-1 secretion levels starting between 6 and 16 h after hypoxic onset, depending on the investigated cell line [14,26,27].

Taken together, these data show for SCCHN cell lines, that PAI-1 mRNA is induced very rapidly upon hypoxic exposure (2 h) [17] with a short delay in PAI-1 expression (BHY and FaDu: 4 h) and an additional lag of some hours in PAI-1 secretion (FaDu: 8 h; BHY: 12 h). The study by Fink *et al*. [14] on the human hepatoma cell line, HepG2, confirms these results by demonstrating a lag in PAI-1 protein synthesis behind the transcriptional up-regulation. This time-lapsed hypoxic induction of PAI-1 mRNA, cellular protein and secreted protein can be explained by the sequence of RNA transcription, translation and secretion.

Here, we report for the first time that *in vitro *hypoxia significantly up-regulates cellular and secreted PAI-1 protein levels after short exposure times (4 – 8 h) and continues up to 24 h in two SCCHN cell lines. Additionally, we have shown an analogous PAI-1 up-regulation by hypoxia in both cell lines, BHY and FaDu. Therefore, our data suggest that *in vivo *already short periods of hypoxia, e.g. caused by the closure of individual capillaries or by temporary reduction in blood flow (= acute hypoxia), might be sufficient to up-regulate PAI-1 in SCCHN. Regarding radioresistance, acutely hypoxic cells are believed to be a more serious problem than chronically hypoxic cells [28]. Therefore, acute hypoxia might be harmful in two ways; on the one hand by causing radioresistance and on the other hand by increasing PAI-1 levels, which most likely leads to a more aggressive phenotype of the surviving radioresistant cells.

An association between high PAI-1 tumour tissue levels and poor outcome has been observed in many clinical studies for different tumour entities, so that PAI-1 has become an established prognostic marker [7-9]. As PAI-1 is a secreted protein, it would be easier to measure PAI-1 levels in the plasma of patients. Therefore, we compared PAI-1 expression and secretion levels during increasing lengths of hypoxic exposure and found a strong correlation between intracellular (Fig. 3A and 3B) and secreted (Fig. 3C and 3D) PAI-1 levels *in vitro*. *In vivo*, no studies exist on SCCHN, however, a clinical study on breast cancer patients found no correlation between PAI-1 levels in tumour tissue and plasma [29]. The authors assume that this discrepancy might be due to inconsistent blood sampling and/or processing. Nevertheless, in a small group of head and neck cancer patients, a relationship between increased PAI-1 serum levels and tumour hypoxia was detected [17]. As high amounts of PAI-1 are released from platelets upon coagulation, PAI-1 determination in blood plasma instead of serum probably would even have shown greater differences between more and less hypoxic tumours. Taken together, this suggests that PAI-1 plasma levels might indirectly reflect the hypoxic status of a tumour and implies that PAI-1 tumour and plasma levels could also correlate *in vivo*. Therefore, *in vivo *studies on SCCHN elucidating the correlation between tumour hypoxia, PAI-1 expression in tumour tissue and PAI-1 plasma levels are promising, but should be performed under standardized blood sampling and processing conditions.

Hypoxic cells require 2.5 – 3 times the radiation dose to produce the same level of cell kill as well-oxygenated cells. Therefore, radiation induced reoxygenation is intended to render hypoxic tumour cells more radiosensitive during fractionated radiotherapy. Indeed, Suzuki *et al*. [30] demonstrated a significantly better local control for patients with reoxygenated cervical cancer. However, Dietz *et al*. [31] showed that a higher degree of tumour reoxygenation during the initial course of chemoradiation or radiotherapy is associated with a poor outcome in head and neck cancer patients. This phenomenon could be due to increased levels of harmful proteins, upon reoxygenation, e.g. proteins involved in tumour invasion and metastasis such as PAI-1. As PAI-1 functions outside the cell, secreted PAI-1 is especially crucial in promoting tumour progression. Most importantly, our results indicate that reoxygenation leads to decreased PAI-1 secretion (Fig. 4C and 4D). Therefore, reoxygenation seems to be favourable not only with regard to increased radiosensitivity, but also with regard to reduced PAI-1 levels.

We are the first to demonstrate that irradiation induces PAI-1 expression and secretion in SCCHN cell lines (Fig. 5). Our results confirm previous *in vitro *studies showing enhanced PAI-1 transcription and expression after ionizing radiation in non-transformed rat tubule epithelial cells [21] and increased PAI-1 transcription in human hepatoma cells [22]. In agreement with these *in vitro *results, Moeller *et al*. [20] observed enhanced PAI-1 expression *in vivo *in irradiated murine breast tumours compared to non-irradiated tumours. As *in vitro *and *in vivo *studies have also demonstrated HIF-1α induction by radiation [20,32], we speculate that radiation induced PAI-1 up-regulation might occur via HIF-1α.

## Conclusion

In summary, our data suggest that both, short-term (~4 – 8 h) and long-term (~20 – 24 h) hypoxic exposure could increase PAI-1 levels in SCCHN *in vivo*. Additionally, our results show that not only hypoxia but also ionizing radiation induces PAI-1 expression and secretion in SCCHN cell lines. This implies that radiation therapy, which is a common treatment modality for the frequently hypoxic head and neck tumours, could lead to enhanced PAI-1 expression and secretion *in vivo*. On the other hand, reoxygenation of hypoxic tumour cells during fractionated radiotherapy could be favourable by counteracting the increased PAI-1 levels.

Therefore, future studies investigating the mechanisms (e.g. reactive oxygen species, HIF) behind hypoxic- and radiation-induced up-regulation of PAI-1 should help to develop strategies (antioxidants, HIF inhibitors) to prevent increased PAI-1 expression and secretion and thereby improve the outcome after radiation therapy.

## Abbreviations

DFO: desferroxamine

ECM: extracellular matrix

ELISA: enzyme-linked immunosorbent assay

HIF: hypoxia inducible factor

HRE: hypoxia responsive element

PAI-1: plasminogen activator inhibitor type-1 (PAI-1)

uPA: urokinase-type plasminogen activator

SCCHN: squamous cell carcinoma of the head and neck

## Competing interests

The author(s) declare that they have no competing interests.

## Authors' contributions

DS has designed the study, performed all experiments (except ELISA) and written the manuscript. CB participated in the drafting of the manuscript and in interpretation of the data. AG and FS performed the ELISA experiments and provided the PAI-1 antibody. MP aided in design of the experiments and contributed substantially to the final version of the paper. KM assisted in design and implementation of experiments (ELISA, IF). LDS contributed to the conception of the study and acquired funding from the Dr. Mildred-Scheel-Stiftung. MM participated in design of the study and provided general supervision of the research. All authors revised the manuscript critically and approved the final version.

## Pre-publication history

The pre-publication history for this paper can be accessed here:


